# Young Children and Adults Show Differential Arousal to Moral and Conventional Transgressions

**DOI:** 10.3389/fpsyg.2020.00548

**Published:** 2020-04-17

**Authors:** Meltem Yucel, Robert Hepach, Amrisha Vaish

**Affiliations:** ^1^Department of Psychology, University of Virginia, Charlottesville, VA, United States; ^2^Research Methods in Early Child Development, Leipzig University, Leipzig, Germany

**Keywords:** moral development, moral norms, conventional norms, affect, pupillometry

## Abstract

From a young age, children understand and enforce moral norms, which are aimed at preserving the rights and welfare of others. Children also distinguish moral norms from other types of norms such as conventional norms, which serve to ensure coordination within social groups or institutions. However, far less is known about the mechanisms driving this differentiation. This article investigates the role of internal arousal in distinguishing moral from conventional norms. In a between-subjects design, 3-year-olds (*n* = 32), 4-year-olds (*n* = 34), and undergraduate students (*n* = 64) watched a video of either a moral norm violation (e.g., destroying another person’s artwork) or a conventional norm violation (e.g., playing a game wrong). Participants of all age groups showed differential physiological arousal (pupil dilation) to moral and conventional norm violations. Participants of all age groups also attended significantly more to the victim of the moral transgression than the bystander in the conventional transgression. Further, this differential attention to the victim/bystander positively correlated with the change in participants’ phasic pupil dilation to the norm violation. This is the first evidence that differences in internal arousal co-occur with (and possibly contribute to) the distinction that even young children draw between moral and conventional norms.

## Introduction

Human group living and cooperation rely heavily on social norms that regulate how one ought to behave (Boyd and Richerson, 2009; Killen and Smetana, 2015). These norms are often enforced by adults—and even young children—to ensure fair treatment of others and harmonious group living by protesting, punishing, or tattling on norm violators (Rakoczy et al., 2008; Balafoutas and Nikiforakis, 2012; Gummerum and Chu, 2014; Riedl et al., 2015; Yucel and Vaish, 2018).

However, not all norms are treated equally. Adults and even young children distinguish between moral norms, which are aimed at preserving the rights and welfare of others (e.g., not causing unprovoked harm or not stealing from others), and conventional norms, which serve to ensure coordination within social groups or institutions (e.g., playing a game correctly or wearing uniforms to school). By 3 years of age, children judge moral norm violations as more serious, moral transgressors as more deserving of punishment, and moral norms as more generalizable across contexts and less contingent on authority (see Smetana et al., 2018, for a review). This distinction is also evident in children’s behaviors. For instance, 3-year-olds protest when a puppet attempts to opt out of moral rules but not conventional rules (Josephs and Rakoczy, 2016), and enforce moral rules on both in- and out-group members but enforce conventional rules selectively on in-group members only (Schmidt et al., 2012; see also Liberman et al., 2018). Children thus distinguish moral from conventional norms remarkably early in development (Smetana, 1981; Smetana, 2013; Ball et al., 2017; Turiel and Dahl, 2019).

Although research has extensively documented that children verbally differentiate between moral and conventional norms, less is known about whether affect underlies these early domain distinctions. Most prior work in this area has focused on whether young children recognize the criteria that are believed to form the basis of these distinctions. In this “social domain” perspective, children’s criterion judgments about norm transgressions and their justifications for their judgments are used to demonstrate that children have moral concepts and recognize distinctive features of morality (see Smetana et al., 2014; Ball et al., 2017). Moral norms function to prevent harm and promote welfare and justice, and thus, children recognize that in contrast to conventional norms, moral norms are obligatory, generalizable, and unalterable. Importantly, these criterion judgments and justifications are fundamentally cognitive in nature: They reflect children’s reasoning and conceptualization of the social world.

There is, however, a growing recognition of the role of emotions and arousal in children’s and adults’ domain distinctions. Social domain theorists propose that children’s own emotional responses and their observation of others’ emotional responses to norm transgressions contribute to the development of their domain distinctions (Turiel and Killen, 2010). For example, children develop a concept of a moral norm against harming others by coming to appreciate which actions cause others harm, an understanding that emerges by empathizing with those who are harmed as well as experiencing the pain of being harmed oneself (Wainryb et al., 2005; Ball et al., 2017). Caregivers also respond differently to moral versus non-moral transgressions. For instance, parents physically intervene more, give more harm-based reasons, and focus more on the victims of harm when enforcing moral norms compared to conventional norms (Dahl and Campos, 2013), and mothers use more angry vocalizations in response to moral transgressions than non-moral transgressions (Dahl et al., 2014). In turn, infants associate firm vocalizations more with moral transgressions than other types of transgressions (Dahl and Tran, 2016). Domain theorists argue that these distinct social interactions and experiences help children construct an understanding of social domains such that by around 3 years of age, they reliably differentiate the different domains.

Emotivists propose a more primary role for the emotions in the moral–conventional distinction. Haidt (2001) prominently proposed that people’s moral judgments are based primarily on affective intuitions and that conscious deliberation primarily serves to produce *post hoc* rationalizations for the intuitive, emotional reactions that occur first. Nichols (2002, 2004) further emphasized the role of affect in the distinctions we make between moral and conventional norms. Specifically, he argued that people (including young children) have a “Normative Theory,” or a nascent understanding of what is acceptable and what is prohibited. When this “Normative Theory” detects a norm violation, and this violation is affectively charged, it is perceived to be a moral norm violation, whereas when the violation is not backed by affect, it is considered non-moral. Indeed, Nichols (2002) showed that if conventional norms become coupled with affect (e.g., disgust), adults no longer perceive them as conventional norms and instead perceive them as belonging to the moral realm, and thus judge them as less permissible, more serious, and more contingent on authority than affect-neutral conventional violations. This effect is also evident in development: by the age of 7 years, children moralize novel rule violations and judge them as more wrong when those rules are backed by affect-laden testimonies (Rottman and Kelemen, 2012; Rottman et al., 2017). These findings offer partial support to Nichols’s account that affect is central to and forms the basis of adults’ and children’s domain distinctions.

Despite the disagreement across theories on the role they prescribe to affect, the aforementioned approaches all agree that affect is involved in the moral/conventional distinction. For example, observational research examining naturalistic responses to transgressions shows that young children respond more emotionally to moral transgressions than conventional or personal transgressions (Nucci and Turiel, 1978; Smetana, 1984, 1989; Nucci and Weber, 1995; Killen and Smetana, 1999). Although 3-year-olds (and in some cases, even 2-year-olds) seem to react more emotionally to moral transgressions, these prior studies were not experimental and thus did not permit causal conclusions. Furthermore, these studies assessed children’s emotional expressions, which may underestimate sensitivity to transgressions in younger children who may experience internal arousal that is not displayed overtly. Surprisingly, no experimental work to date has shown whether affect is involved when children *first* begin to make the distinction between moral and conventional norms around 3 years of age. It thus remains unknown whether affect in fact underlies the psychological distinction young children draw between moral and non-moral norms, as both social domain theorists and emotivists propose. Absent evidence for this claim, we must consider the alternative possibility that affect only enters the picture later in development, that is, it is layered on top of moral judgments that initially emerge as a result of more deliberate processes. Moreover, establishing the role of affect in early development is a foundational step toward understanding *how* it is involved. In particular, it holds promise for addressing the tension in the literature about whether affective responses to norm transgressions result from or give rise to children’s domain distinctions. Our goal in the present study was to take this foundational step.

Notably, one recent study did broach this issue in the context of children’s direct involvement (Hardecker et al., 2016). This study revealed that 5-year-old but not 3-year-old children showed more overt agitation (such as anger, annoyance, or yelling at the transgressor) to moral than to conventional transgressions. Although this hints that affect may not be involved when children first begin to distinguish moral from conventional transgressions at age 3—challenging findings from prior observational studies—it is also possible (as the authors acknowledge) that the task was too demanding for this younger group. For instance, perhaps 3-year-olds in this study did experience greater internal arousal to moral than conventional transgressions, but this did not manifest itself in their overt behaviors. A more sensitive measure of internal arousal could provide evidence of distinct affective responses to moral versus conventional transgressions even at this young age.

In the present study, therefore, we asked whether moral transgressions elicit different levels of internal arousal at the age when children first begin to make the moral–conventional distinction (3 years) or only after children have had some experience making the moral–conventional distinction on more conceptual grounds (4 years). Furthermore, we asked whether any differences in internal arousal that children show are comparable to those shown by adults in order to chart the developmental trajectory of the affective responses to norm transgressions.

In contrast to previous work, which relied on children’s behavioral or verbal measures of affect (Arsenio and Ford, 1985; Hardecker et al., 2016), we assessed children’s and adults’ internal affective arousal via changes in their pupil dilation. Human pupils dilate in response to affectively charged images and audio stimuli (Partala and Surakka, 2003; Bradley et al., 2008; Henderson et al., 2014). Greater internal arousal corresponds to increased pupil dilation, which can be measured via slower (tonic) changes and more immediate (phasic) changes (Sirois and Brisson, 2014; Hepach and Westermann, 2016). Children’s tonic pupil dilation increases in response to harm-like situations such as seeing others needing help and children anticipating the correct solution to resolve the situation (Hepach et al., 2016; Hepach and Herrmann, 2019).

An important advantage of pupillometry is that, unlike behavioral or verbal measures, which are often limited in terms of the ages with which they can be used, pupil dilation can be measured and interpreted similarly across ages (Hepach and Westermann, 2016; Krüger et al., 2019). Past studies on moral development that have relied on behavioral or verbal measures have often included either young children or adults but have not compared young children’s responses to those of adults (Arsenio and Ford, 1985; Nichols, 2002; Rottman and Kelemen, 2012; Hardecker et al., 2016; Rottman et al., 2017; though see Decety et al., 2012). Our use of pupillometry, on the other hand, allows us to examine internal arousal to norm transgressions across age groups. Specifically, we assessed both tonic and phasic changes in participants’ pupil dilation in response to moral and conventional transgressions. This allowed us to investigate (1) shifts in children’s arousal state (tonic changes in pupil dilation) as well as (2) time-locked immediate changes in pupil dilation in response to viewing key events in the situations presented to children (phasic changes).

The present study addressed two further questions regarding children’s responses to norm transgressions. The first was whether children and adults pay attention to different aspects of a moral versus a conventional norm violation. Prototypical moral transgressions generally involve victims (because they involve harm or injustice), and so they affect other people, whereas conventional transgressions do not directly harm other people (Turiel, 1983; Smetana, 1985). In fact, prior work indicates that children and adults look more at the victims (people or objects) that are the target of harmful actions than they look at the perpetrators (Decety et al., 2012, p. 216). We thus hypothesized that victims of moral transgressions should elicit greater attention than bystanders who are present during (but not harmed by) conventional transgressions (see also Vaish et al., 2009). This differential looking would also importantly demonstrate that, in line with prior work, participants did discriminate between the two types of transgressions. We thus used gaze tracking to measure participants’ looking to the victim in the moral transgression as compared to looking to the bystander in the conventional transgression. Looking time has been long used with infants and children to measure their attention allocation to social stimuli (Aslin, 2007; Gredebäck et al., 2009; Decety et al., 2012). We further assessed whether this differential attention correlates with differences in internal arousal, either because those who focus more on the victim may thereby become more affectively involved in the transgression or, alternatively, those who are more affectively involved in a transgression may attend more to the victim.

A more exploratory question concerned participants’ resource allocation toward the transgressors versus the victim/bystander. Both adults and children show concern and prosocial behavior toward victims of moral transgressions (Vaish et al., 2009; Vaish et al., 2011a; Decety et al., 2012; Leliveld et al., 2012) and punish moral transgressors, such as by taking resources away from them or allocating fewer resources to them (Fehr and Fischbacher, 2004; Leliveld et al., 2012; Reich and Hershcovis, 2015; Smetana et al., 2018). Smetana et al. (2018) found that children allocated fewer resources to moral than conventional transgressors. Moreover, in interview studies, children judge moral transgressions as more punishable than conventional transgressions (Smetana, 1981). It is thus possible that children and adults also allocate more resources to the victim of a moral transgression than the bystander in a conventional transgression, and fewer resources to a moral than a conventional transgressor. To explore these possibilities, we ended the study with a resource allocation task wherein participants could distribute three resources between the transgressor and victim/bystander.

Finally, based on a recent study showing a positive association between changes in children’s arousal (measured via tonic changes in pupil dilation) and their motivation to help (Hepach et al., 2018), we assessed whether participants’ pupil dilation correlated with their resource allocations. Though we did not have strong predictions, we considered that participants (i) may feel more concern for and thus allocate more resources to a victim than a bystander and (ii) may judge moral transgressions more negatively and thus allocate fewer resources to a moral transgressor than a conventional transgressor. Further, participants who showed greater arousal (pupil dilation) to a transgression may have greater prosocial motivation and thus allocate more resources to the victim/bystander. With these experimental and exploratory goals, we aimed to shed light on the affective, attentional, and behavioral aspects of the moral–conventional distinction in early development.

## Materials and Methods

### Participants

Children were 32 3-year-olds (age range: 36–47 months, *M* = 39.78 months, SD = 3.23 months; 15 female) and 34 4-year-olds (age range: 48–58 months, *M* = 51.93 months, SD = 2.51 months; 17 female; see Supplementary Table S1). Ten additional children were tested but excluded due to parental interference (*n* = 3), because they did not provide sufficient pupil data for analyses (*n* = 3), because they wore glasses and could not be recorded by the eye tracker (*n* = 1), because they cried and did not complete the study (*n* = 2), or due to experimenter error (*n* = 1). The sample size was determined as 16 children per cell, based on previous behavioral research on the development of norm understanding (Vaish et al., 2011b; Schmidt et al., 2012; Yucel and Vaish, 2018). Because children were oversampled to account for possible exclusions, we ended up testing and retaining two more 4-year-olds than originally planned. Participants were recruited from a database of parents living in a medium-sized university town in the United States. Of the families that provided information about race (59 out of 66), 78% of the children were White, 6.8% Black, 1.7% Asian, and 13.6% multiracial. Of the parents that provided information about their educational attainment (64 out of 66), 6.3% graduated from high school, 29.7% graduated from college, and 64.1% of the parents graduated from a post-graduate institution. Children received a toy for participating. The procedure was approved by the authors’ institutional review board, and parents consented to their children’s participation.

Adult participants were 64 undergraduate students (age range: 18–28 years, *M* = 19.19 years, SD = 1.44 years) at the university where the study was conducted. We doubled the adult sample size (32 adults per cell) to match 64 children across age groups. All adult participants provided information about their race: 65.6% were White, 10.9% Asian, 6.3% Hispanic, 3.1% Black, and 14.1% multiracial. Participants were compensated with course credit. The procedure was approved by the authors’ institutional review board, and participants provided informed consent prior to participation.

### Setup

Participants were seated approximately 60 cm away from a 24-inch monitor (52 cm × 32 cm) with a resolution of 1,680 × 1,050 pixels. An eye tracker (Tobii model X120; Tobii Technology, Stockholm, Sweden) with a sampling rate of 60 Hz was positioned below the screen. Stimuli were presented, and participants’ eye gaze and pupil diameter were recorded using Tobii Studio (version 3.3.0; Tobii Technology, Stockholm, Sweden). The system was calibrated to participants’ eyes using a five-point calibration procedure. To ensure that luminance was similar across participants, all participants were tested in the same room in the lab under the same lighting conditions. From where participants sat in the room, the luminosity was measured at 80 lux when facing the screen that was turned off. The screen was turned on at least 20 min prior to participants’ arrival.

### Procedure and Design

Testing took place in a child development lab at a university. In a reception area, the study was described to parents, and parents gave consent for their child’s participation. Similarly, adult participants were informed about the study and gave consent before participating. Once children were comfortable with the setting, the experimenter (who was not featured in the videos that the child would later watch) and the child entered the study room. The child sat in a high chair (see Supplementary Figure S1). The experimenter sat behind a barrier and did not interact with the child unless the child was not paying attention, in which case the experimenter pointed at the screen and said, “Look.” Adult participants sat either in a small chair or on a cushion, depending on their height.

**FIGURE 1:**
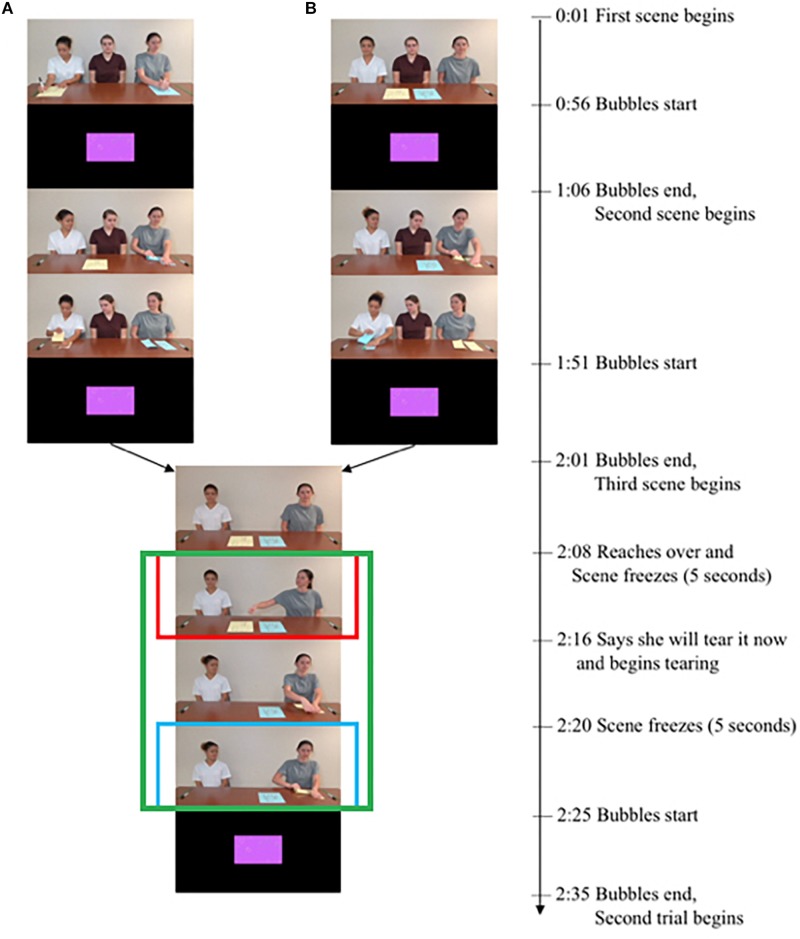
Timeline of **(A)** Moral condition: two actors each drew a picture, and the transgression began when one actor reached over to the other’s picture (2:08); and **(B)** Conventional condition: two actors were instructed that the game involves tearing pictures using a ruler (or squashing clay sculptures using a block), and then one actor transgressed by using her hand to tear/squash the picture/block (2:20). Note that the final (transgression) scene was identical across conditions, but its interpretation varied based on what had transpired previously. In the Moral condition, pupil dilation was analyzed during the first freeze frame after the moral transgression began (2:08–2:10; red rectangle). In the Conventional condition, pupil dilation was analyzed during the first freeze frame after the conventional transgression began (2:20–2:22; blue rectangle). Gaze duration was analyzed from 2:08 to 2:25 (green rectangle). The individuals in the image provided written, informed consent for the publication of the image.

We randomly assigned participants to either the Moral or Conventional condition (between-subjects). Each condition consisted of two trials: one featured a drawing activity, and the other featured a clay sculpting activity (order counterbalanced across participants). In both conditions, participants watched a 5 min video of two female actors engaging in an art activity (drawing or clay sculpting) and one actor violating either a moral rule (destroying another actor’s picture or sculpture with her hands) or a conventional rule (destroying a picture or sculpture with her hands instead of following the game rule of using a ruler/block; see Figure 1). After watching the videos, participants were asked to distribute three flowers between the two actors (the transgressor or the victim/bystander) from the video. The entire session lasted approximately 10 min.

#### Videos

Each video consisted of three action scenes: rule introduction, rule following, and rule transgression (see Figure 1 for the timeline and Supplementary Material for the detailed video script). In the Moral condition, the narrator introduced the rule that drawings/sculptures belonged to the actors and only the actor who made the drawing/sculpture was allowed to destroy it. In the Conventional condition, the narrator introduced the rule that drawings/sculptures (which did not belong to anyone) may only be torn/squashed using the ruler/block (by putting the ruler on top of the drawing and tearing along the ruler, or by putting the block on top of the sculpture and squashing it). In both conditions, participants then watched the rule-following scene, in which the first actor acted according to the rule (destroying the objects with the ruler/block in Conventional and destroying her own artwork in Moral). The narrator then reiterated the rule. The rule-introduction and rule-following scenes were kept as similar as possible across trials and conditions.

The final scene (rule transgression) was identical across conditions: it featured the transgressor reaching toward the piece of paper (drawing activity) or clay sculpture (clay activity) that lay in front of the other actor and then tearing the paper or destroying the clay sculpture. The scene paused for 5 s after the transgressor reached for the paper/clay sculpture, to ensure that participants were paying attention to which paper/clay the transgressor was reaching toward. In the Moral condition, the drawing or sculpture that the transgressor destroyed belonged to the second actor sitting next to her, and the transgressor thus violated the moral rule. In the Conventional condition, the transgressor violated the rule by using her hands instead of the ruler/block to destroy the picture/sculpture. For both conditions, the transgression scene ended before the transgressor completed the tearing or squashing action. The victim/bystander watched the transgression neutrally and showed no emotional response at all.

**FIGURE 2:**
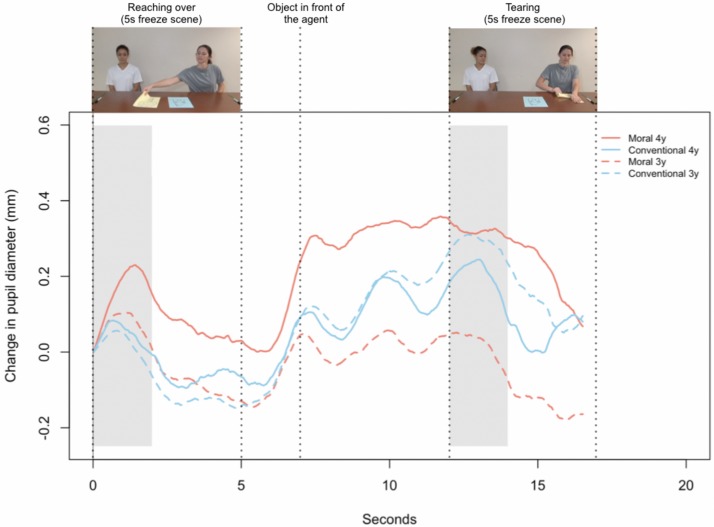
Changes in pupil diameter for 3- and 4-year-olds, starting with the transgressor reaching over until the scene ends. Gray segments indicate the 2 s of still frame that were used for analyses: reaching over in the Moral condition and tearing (squashing) in the Conventional condition. The individuals in the image provided written, informed consent for the publication of the image.

Note that the final (transgression) scene was identical across conditions. We initially intended to compare pupil dilation at identical sections of the scene: when the transgressor begins to destroy the object. We subsequently realized, however, that the moral transgression in fact starts earlier than the conventional transgression: the moral transgression starts as soon as the transgressor reaches over and touches the other person’s belonging [2 min 8 s (or 2:08) to 2:10; see Figure 1), since this already suggests to the viewer that the transgressor might destroy the other person’s object. On the other hand, when the conventional transgressor reaches over and touches the same object, this action is not yet a transgression since it does not belong to the other person; the conventional transgression only starts later (2:20–2:22), when the transgressor uses her hand rather than using a ruler/block to destroy the object. We thus focused our analyses on the earlier time window for the moral transgression and the later time window for the conventional transgression (see Figure 2).

Following each scene, participants watched an attention-grabbing neutral animation for 8 s (purple bubbles on a black background with music, previously used by Hepach et al., 2016). This bubble scene was used to engage participants with the video and to measure tonic changes in pupil dilation. Trial order, role distribution (which actor played the transgressor and the victim/bystander), and whether the transgressor sat on the left or right of the screen were fully counterbalanced across conditions and participants.

#### Distribution of Resources

Following the completion of the eye-tracking task, participants were seated at a different table for a flower distribution task (based on Vaish et al., 2011a). The experimenter instructed participants as follows: “I’m going to see Sally (transgressor) and Katie (victim/bystander) soon and can bring them something from you. Look. I have a box for each of them.” The experimenter then put two boxes in front of the participant, one with a picture of Sally and the other with a picture of Katie, on the same sides (left or right) that the actors sat on in the videos. The experimenter then said: “I have these three flowers. You can hand these out as you want, and then I’ll take the boxes to them.” If the participant did not distribute immediately, the experimenter said, “You can put the flowers in the boxes.” After the participant distributed the flowers, the experimenter recorded the number of flowers distributed to each actor and asked the participant why they gave Sally or Katie more flowers.

### Data Processing and Statistical Analyses

The raw data were exported from Tobii Studio as text files. Given the sampling frequency, 60 samples were collected per second. Each sample contained the following information organized in columns: recording time stamp, gaze event type, and for each eye, X- and Y-gaze-position as well as pupil diameter in millimeters. Fixations were defined within Tobii Studio using the Velocity-Threshold Identification (I-VT) standard fixation filter with a window length set to 20 ms and a velocity threshold set to 30°/s. The text files were read into R (R Core Team, 2017; version 3.4.0). We wrote scripts that automated the processing of eye data following previously established algorithms (Hepach et al., 2016). The statistical analyses for pupil dilation were carried out in R (R Core Team, 2017; version 3.4.0), focusing on the first test trial (similar to Hepach et al., 2018; Vaish et al., 2018). All reported statistical tests and the associated *p*-values from pairwise comparisons are two-tailed.

#### Pupil Dilation: Tonic Changes

We calculated the change in children’s pupil dilation from the first neutral (bubble) clip after rule introduction (baseline: 0:56–1:06) to the neutral clip after the rule following (process measure: 1:51–2:01) and the change from the neutral clip after the rule following to the neutral clip after the rule transgression (transgression measure: 2:25–2:35). We filtered data to remove the upper tercentile of sample-to-sample differences and linearly interpolated gaps not exceeding 70 ms. Data for each participant were pre-processed using previously established algorithms (Hepach et al., 2016).

#### Pupil Dilation: Phasic Changes

We compared changes in pupil size during the still frame following each transgression (Moral: 2:08–2:10, immediately after the transgressor touches the victim’s object; Conventional, 2:20–2:22, immediately after the transgressor begins destroying the object; we chose these different time windows because the moral transgression began earlier than the conventional transgression; see Figures 1, 2). This comparison assessed whether participants in the Moral condition showed a different physiological arousal compared to participants in the Conventional condition. Additionally, to verify that participants did indeed perceive the act of touching the other’s belonging as a transgression in the Moral more than in the Conventional case, we compared changes in pupil size during the still frame immediately after the transgressor touches the object in both Moral and Conventional conditions (2:08–2:10). We expected that participants would show different levels of arousal upon seeing the actor touch the object in the Moral condition compared to those in the Conventional condition.

Data for each participant were pre-processed using previously established algorithms (Hepach et al., 2016). We filtered data to remove the upper tercentile of sample-to-sample differences and linearly interpolated gaps not exceeding 70 ms. After pre-processing the data, we calculated change scores in pupil dilation by subtracting from each sample within the 2 s time window the average of the seven samples (corresponding to approximately 100 ms) immediately preceding the time window (see also Vaish et al., 2018). For the resulting baseline-corrected changes in pupil size, the variances were similar for Moral and Conventional conditions, *F*(5,116) = 0.856, *p* = 0.513. We calculated an ANOVA with age group (3-year-olds, 4-year-olds, and adults) and condition (Moral versus Conventional) as a categorical predictor variable and the change in pupil size as the dependent measure. The statistical analyses were carried out in R (R Core Team, 2017; version 3.4.0).

#### Duration of Looking

**FIGURE 3:**
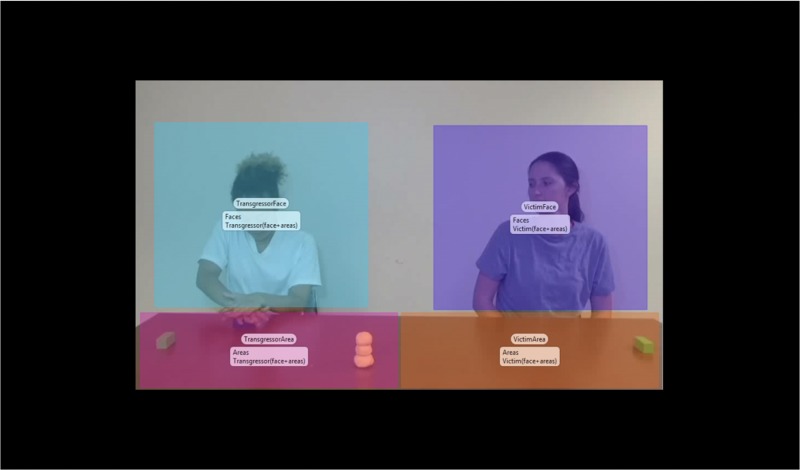
Areas of interest (AOIs) created using Tobii Studio (version 3.3.0; Tobii Technology, Stockholm, Sweden). Transgressor AOI encompassed the transgressor (Sally) and the area in front of the transgressor (10.51% of the total screen). Victim or bystander AOI encompassed the victim/bystander (Katie) and the area in front of the victim/bystander (10.51% of the total screen). The individuals in the image provided written, informed consent for the publication of the image.

We defined two areas of interest (AOIs) within Tobii Studio. One encompassed the transgressor as a whole including the area in front of the transgressor, and the other focused on the victim/bystander as a whole including the area in front of the victim/bystander (Figure 3). We focused on the total fixation duration from when the transgressor began tearing (2:08) until the scene ended (2:25) because we were interested in capturing the differences in how much attention participants paid to different characters involved in moral and conventional transgressions as the transgression took place. Participants’ looks to the victim/bystander were calculated as: the number of samples belonging to a fixation mapped onto the transgressor’s AOI divided by the duration of the scene (17 s) and multiplied by 100 to result in percentage values. We calculated an ANOVA with age group (3-year-olds, 4-year-olds, and adults) and condition (Moral versus Conventional) as categorical predictor variables and the proportion of looking time to the transgressor as the dependent measure. The statistical analyses were carried out in SPSS.

#### Distribution Task

We measured how many of the three flowers participants distributed to the transgressor versus the victim/bystander. We calculated a 2 × 3 ANOVA with condition (Moral versus Conventional) and age (3-year-olds, 4-year-olds, and adults) as categorical independent variables and the number of flowers distributed to the transgressor as the dependent variable. The statistical analyses were carried out in SPSS. Levene’s test indicated equal variances across conditions, *F* = 0.51, *p* = 0.475.

#### Individual Differences

We measured how participants’ phasic pupil dilation during the transgression still frames (Moral: 2:08–2:10; Conventional, 2:20–2:22; see Figures 1, 2) correlated with participants’ looks to the victim/bystander (2:08–2:25) and with the number of flowers distributed to the transgressor. We also measured how participants’ tonic pupil dilation during the neutral (bubble) scene following the transgression (2:25–2:35; see Figure 1) correlated with participants’ looks to the victim/bystander (2:08–2:25) and with the number of flowers distributed to the transgressor. The statistical analyses were carried out in SPSS.

## Results

### Duration of Looking

**FIGURE 4:**
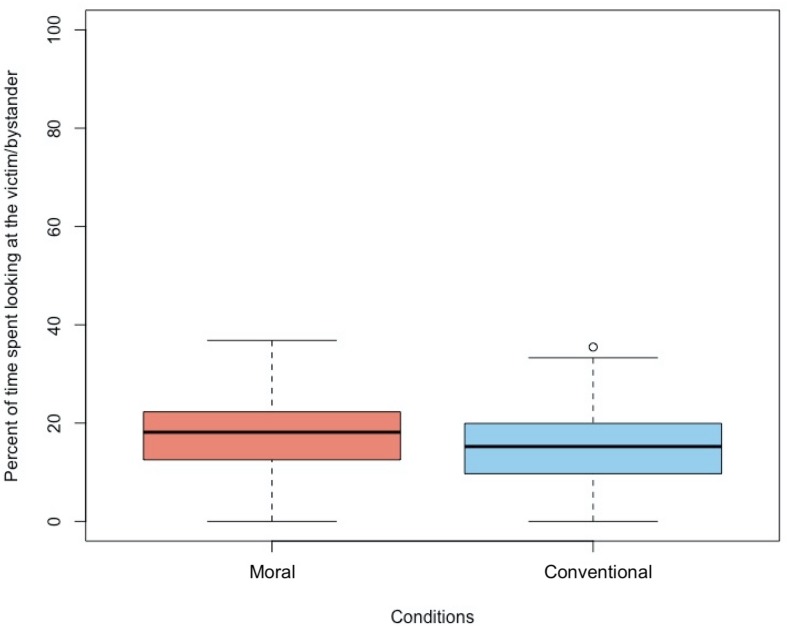
Percent of time spent attending to the victim in the Moral condition versus the bystander in the Conventional condition.

Across conditions, participants attended for similar amounts of time to the moral transgression scene (*M* = 11.15 s, *SD* = 3.49) as the conventional transgression scene (*M* = 11.50 s, *SD* = 3.19), *t*(128) = 0.60, *p* = 0.551. However, as predicted, across ages, participants looked for a significantly greater proportion of time at the victim during the moral transgression (*M* = 17.22%, *SD* = 8.04) than at the bystander during the conventional transgression (*M* = 14.64%, *SD* = 7.91), *F*(1, 124) = 6.19, *p* = 0.014, $\eta_{p}^{2}=0.05$ (see Figure 4; also see Supplementary Table S2 for cell means per condition). This analysis of the proportion of time spent looking at the victim/bystander did not reveal a main effect of age, *F*(2,124) = 2.44, *p* = 0.091, $\eta_{p}^{2}=0.04$, or an interaction between age and condition, *F*(2,124) = 2.32, *p* = 0.103, $\eta_{p}^{2}=0.04$.

### Pupil Dilation: Phasic Changes

**FIGURE 5:**
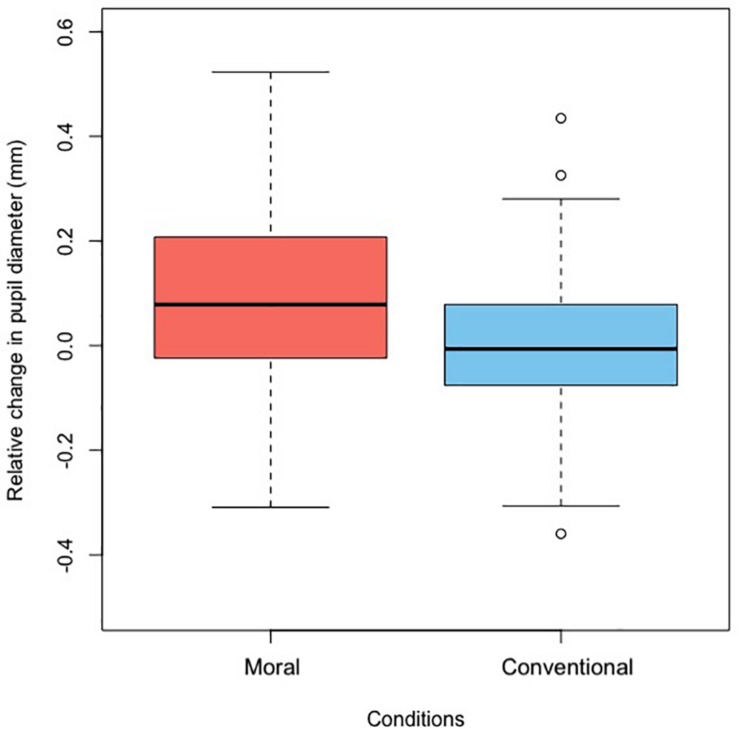
Phasic changes in pupil dilation, collapsed across age groups. Baseline for this graph was taken 100 ms before the respective freeze frame, i.e., 100 ms before the first freeze frame (reaching over) in the Moral condition and 100 ms before the second freeze frame (tearing/squashing) in the Conventional condition.

In support of the hypothesis that moral transgressions elicit a different pattern of arousal compared to conventional transgressions, we found that participants’ pupil size increased more during the moral (*M* = 0.10, *SD* = 0.15) than the conventional transgression (*M* = 0.01, *SD* = 0.14), *F*(1,112) = 11.50, *p* = 0.001, η*^2^* = 0.09 (see Figure 5; also see Supplementary Table S2). There was no interaction between age and condition, *F*(2,112) = 0.97, *p* = 0.381, η^2^ = 0.01.

As predicted, we also found that during the rule-transgression scene, participants’ pupil size increased more during the still frame after the actor touched the drawing or sculpture in the Moral condition (2:08–2:10) than in the Conventional condition, *F*(1,111) = 6.91, *p* = 0.010, η^2^ = 0.06 (see Supplementary Table S2). There was no effect of age and no interaction between age and condition, *p* = 0.058 and *p* = 0.435, respectively.

### Pupil Dilation: Tonic Changes

To investigate whether the effects on participants’ phasic changes in pupil dilation were a result of their tonic arousal state changing, we additionally analyzed the tonic changes in pupil dilation. We found that tonic changes in response to the rule following varied as a function of condition and age group, *F*(2,99) = 4.37, *p* = 0.015, η^2^ = 0.08 (see Supplementary Table S2). Both 3-year-olds and 4-year-olds showed greater pupil dilation after the conventional rule was followed compared to after the moral rule was followed: *M*_3__yo–conventional_ = 0.04 (*SD*_3__yo–conventional_ = 0.06), *M*_3__yo–moral_ = -0.003 (*SD*_3__yo–moral_ = 0.05), *M*_4__yo–conventional_ = 0.04 (*SD*_4__yo–conventional_ = 0.04), *M*_4__yo–moral_ = 0.006 (*SD*_4__yo–moral_ = 0.05). On the other hand, there was no difference for the adult participants when the conventional rule was followed (*M* = −0.003, *SD* = 0.06) compared to the moral rule (*M* = 0.02, *SD* = 0.07). We did not find systematic changes in participants’ tonic arousal after they viewed the transgressions, *F*s < 3, η^2^ < 0.06.

### Distribution Task

**FIGURE 6:**
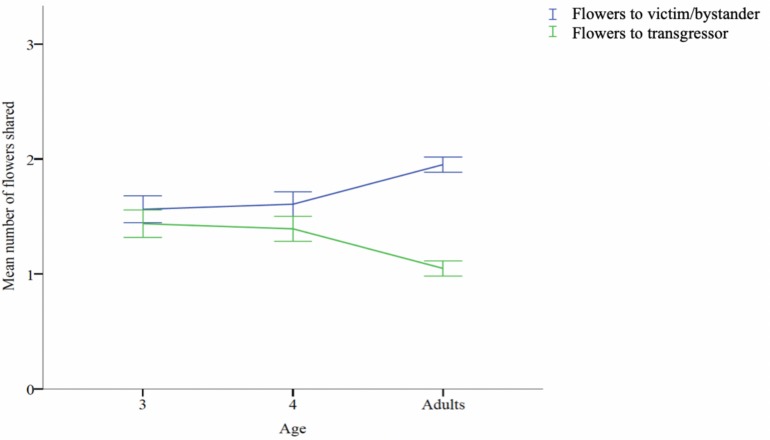
Mean number of flowers shared with the transgressor versus the victim/bystander by each age group. Error bars indicate standard error.

All participants distributed all three flowers between the two actors. The 2 × 3 ANOVA did not reveal a significant condition × age interaction (*p* = 0.919) or a main effect of condition (*p* = 0.179) but did reveal a significant main effect of age on the number of flowers shared with the transgressor, *F*(2,116) = 6.02, *p* = 0.003; $\eta_{p}^{2}=0.094$ (see Figure 6; also see Supplementary Table S2 for cell means). Follow-up pairwise comparisons (with Bonferroni correction) revealed significant differences between adults’ and children’s flower sharing behavior, such that adults distributed fewer flowers to the transgressor (*M* = 1.05, *SD* = 0.53) than 3-year-olds (*M* = 1.44, *SD* = 0.67) as well as 4-year-olds (*M* = 1.39, *SD* = 0.57), *p* = 0.009, *d* = 0.65, and *p* = 0.034, *d* = 0.62, respectively. Three- and 4-year-olds did not differ on the number of flowers they distributed to the transgressor (or the victim/bystander, since they are perfectly and inversely correlated), *p* = 1.00. Paired-samples *t*-tests were conducted to compare the number of flowers that participants in each age group shared with the transgressor versus the victim/bystander. Adults shared significantly fewer flowers with the transgressor (*M* = 1.05, *SD* = 0.53) than the victim/bystander (*M* = 1.95, *SD* = 0.53); *t*(61) = 6.77, *p* < 0.001, *d* = 0.86. However, neither 3- nor 4-year-old children distributed significantly different amounts to the transgressor versus the victim/bystander, both *p*s > 0.3.

### Individual Differences

Participants’ duration of looking to the victim/bystander was positively correlated with the change in their phasic pupil dilation to the transgression (reaching in Moral and tearing/squashing in Conventional), *r*(118) = 0.19, *p* = 0.043, *R*^2^ = 0.04. That is, the longer participants looked at the victim/bystander during the transgression, the greater was their pupil dilation to the transgression. However, the number of flowers participants shared with the transgressor (or the victim/bystander, since they are perfectly and inversely correlated) did not correlate with their gaze duration to the victim/bystander or their phasic pupil dilation, *p* = 0.586 and *p* = 0.135, respectively. Results for these correlations were similar when Moral and Conventional conditions were analyzed separately. Participants’ tonic internal arousal to the transgression did not correlate with the duration of looking to the victim/bystander or the number of flowers participants shared with the transgressor, both *p*s > 0.8.

## Discussion

Norms are integral to human group living and cooperation, and are followed and enforced from early in human ontogeny (Rakoczy et al., 2008; Boyd and Richerson, 2009; Riedl et al., 2015; Yucel and Vaish, 2018). But not all norms are treated equally. Prior behavioral work has consistently shown that children as young as 3 years of age can distinguish moral norms from conventional norms and treat moral violations as more serious and moral transgressors as more deserving of punishment (Smetana et al., 2018). Yet far less is known about the mechanisms underlying this distinction in early development, and particularly about whether affect accompanies the distinction from early in development.

The present study was designed to investigate whether observing moral transgressions elicits different degrees of internal arousal compared to conventional transgressions and whether it does so even at the youngest age at which children draw a conceptual distinction between moral and conventional transgressions (3 years). In line with our expectations and prior work (Nucci and Turiel, 1978; Smetana, 1984, 1989; Nucci and Weber, 1995; Killen and Smetana, 1999), we found that adults showed greater pupil dilation immediately after viewing moral violations than conventional violations (phasic changes). The important and novel finding was that this effect also emerged among children: both 3- and 4-year-olds showed greater pupil dilation when viewing moral violations than conventional violations. This is, to our knowledge, the first evidence that when children first begin to make the conceptual distinction between moral and conventional violations (around 3 years of age), they are also differentially aroused by such violations. Differential affective arousal is thus present at least as early in ontogeny as the conceptual differentiation.

The one prior study to examine this question with preschool-age children found evidence for greater emotional behaviors (such as anger or yelling) to moral than to conventional transgressions among 5-year-old children but not 3-year-old children (Hardecker et al., 2016). However, the absence of evidence among 3-year-olds in that study may have been due to the task or behavioral measures being too demanding. Here, we used a more sensitive measure of internal arousal (pupillometry), and found evidence of greater internal arousal (phasic changes) to moral transgressions even at this young age. As this arousal difference also emerged among older children (4-year-olds) as well as adults, our findings indicate that moral norm violations induce greater internal arousal than conventional violations throughout development. These results, in conjunction with existing behavioral data with older children and adults that demonstrate disparate affective responding to moral compared to conventional norm transgressions (Nichols, 2002; Rottman and Kelemen, 2012; Hardecker et al., 2016), offer strong support that affective arousal is an important and constant feature of the moral–conventional distinction and warrants greater attention.

More crucially, the present phasic results provide the first empirical support for existing proposals that argue that moral transgressions elicit differential affective arousal compared to conventional transgressions from very early in ontogeny and that this affective differentiation informs the developing moral–conventional distinction in important ways (Arsenio and Ford, 1985; Nichols, 2002; Dahl and Tran, 2016; Smetana et al., 2018). With this fundamental piece in place, future research can begin to examine *how* affect is involved and, in particular, to tease apart long-debated questions about whether this emotional differentiation *results* from children’s domain distinctions, as proposed by social domain theorists (Smetana, 2013), or whether it *gives rise* to children’s domain distinctions, as proposed by theorists who place emotions at the heart of the moral–conventional distinction (Nichols, 2004).

In addition to arousal differences, we were interested in potential differences in how people allocated their attention to characters involved in transgressions. Because moral violations typically involve victims, whereas conventional violations do not, and based on prior work (Decety et al., 2012), we predicted that participants would look more at the victim in the Moral condition (e.g., out of concern or to check how she will react to her belongings being destroyed; cf. Vaish et al., 2009). This prediction was confirmed, suggesting that both young children and adults see moral violations as having a greater effect on others than conventional violations. Moreover, participants’ phasic pupil dilation was positively correlated with their duration of looking to the victim/bystander. This correlation may reflect that those who attended more to the affected party in a transgression became more affectively involved in the transgression or, alternatively, that those who were more affectively involved in a transgression attended more to the affected party. Teasing these possibilities apart will be an interesting direction for future research.

Although participants’ phasic pupil dilation correlated with their duration of looking to the victim/bystander, their tonic pupil dilation did not. In prior work in which researchers found a positive correlation between children’s tonic pupil dilation and behavior, the experimental manipulation was stronger (through the use of live paradigms that involved children directly), but phasic pupil dilation was more difficult to measure (see Hepach et al., 2018). However, in a screen-based study like ours, in which phasic changes could be measured reliably but the manipulation and direct involvement of participants were weaker, we found a link between phasic pupil dilation and behavior. Moreover, prior work assessed the relation between pupil dilation and prosocial behavior (Hepach et al., 2018), whereas we examined the relation between pupil dilation and looking behavior. Future studies should incorporate both phasic and tonic analyses and examine in more detail how immediate and slow changes in pupil dilation may relate differently to different forms of social and prosocial behaviors.

Finally, we examined participants’ resource allocation toward the transgressor. In prior work, 2.5- and 4-year-olds allocated fewer resources to moral than conventional transgressors (Smetana et al., 2018). We thus expected participants to allocate fewer resources to the moral than conventional transgressor. However, we did not see this pattern in our study. Adults distributed fewer resources to transgressors in general, with no difference between the Moral and Conventional conditions. Thus, adults who witnessed the moral transgression did not seem to give resources as a way to compensate or show empathy for the victim, since they gave a similarly high amount to the bystander in the conventional transgression. Rather, adults may have been motivated to punish transgressors, regardless of type of transgression (Fehr and Fischbacher, 2004). Alternatively or in addition, adults may have been motivated to reward the victim/bystander for not having transgressed. Children of both ages also did not distribute differently between the moral and conventional transgressors. Indeed, their distribution did not differ even between the transgressor and the victim/bystander. It is possible that because the distribution task was at the end of our study and was conducted in a different part of the testing room where the videos were no longer visible, children found it challenging to recall which actor was the transgressor, particularly as they were not themselves involved in the transgression but simply watched videos of third-party interactions. Future work could include a memory check before the distribution task to resolve this potential problem. In sum, although young children responded similarly to adults (physiologically and in their attention allocation), behaviorally, they differed from adults such that they did not show a pattern of punishing transgressors or empathizing with victims/bystanders. This pattern of findings lends support to the use of physiological measures rather than relying solely on behavioral measures, which are often difficult to compare and interpret across ages.

It is worth noting that for our phasic analyses, we compared the first 2 s of the moral transgression to the first 2 s of the conventional transgression. Although we initially set up our study to compare tonic pupil dilation to the identical scene (the neutral bubble scene after the transgressor destroys the object), we subsequently realized that the moral transgression may in fact be perceived as beginning earlier: as soon as the transgressor reaches over and touches the other person’s object. Thus, by the time the final neutral scene begins, it has been 17 s since the moral transgression started but only 5 s since the conventional transgression started. Perhaps because the moral transgression started 12 s earlier than the conventional transgression, the moral arousal may have dampened by the final neutral scene. Indeed, there was no significant difference in participants’ tonic arousal measured during the final neutral scene. For this reason, we modified our analysis time frames to accommodate the different start points and focused on phasic (immediate) changes to those start times instead.

Two other findings from our study supported our reasoning to compare these different start times. First, we reasoned that if the moral transgression was indeed perceived to begin when the transgressor touched the other person’s object (2:08–2:10), whereas the conventional transgression was not yet perceived to begin at this point, then we should see evidence for higher arousal at this time point in the Moral than in the Conventional condition. This was indeed the case: Participants of all age groups were more aroused when the actor touched the object in the Moral than in the Conventional condition. Second, participants’ phasic pupil dilation in response to the transgressions (touching the other’s object in the Moral condition and destroying the object incorrectly in the Conventional condition) positively correlated with the time they spent looking at the victim/bystander throughout the transgression. This indicates that differences in their pupil dilation were not merely a response to superficially different aspects of the scenes but, rather, an index of the distinct ways in which they were interpreting the scenes.

Nonetheless, it could be argued that the distinct pupillary responses across conditions may not arise from participants’ distinct interpretations of the scenes as *moral* versus *conventional* but may instead reflect a lower-level difference between the scenes. Specifically, in the Moral condition, the transgressor reaching for the other person’s object violates the moderator’s instruction to “only work on your own drawing/sculpture.” In the Conventional condition, however, the same action does not constitute a violation, as the moderator in the Conventional case did not provide any instructions in this regard. On this account, the increased internal arousal accompanying reaching in the Moral condition (2:08–2:10) may not be due to moral reasons such as anticipated harm to the victim but, rather, due to violation of instructions in the Moral case but not yet in the Conventional case. Though we acknowledge this alternative possibility, we note that it cannot account for the full set of findings. In particular, the positive correlation between participants’ phasic pupil dilation and their duration of looking to the victim/bystander suggests that the increased arousal to the reaching action in the Moral condition not only was a reaction to an instruction being violated earlier in that condition but also carried some moral (or at least victim-related) relevance. Still, we call for future studies using this methodology to optimize the moral and conventional transgression scenes in order to be able to compare identical sections of the scenes and thus further enhance comparability across transgressions.

In our study, we aimed for visual comparability across conditions by framing an identical action (i.e., tearing a paper or squishing a sculpture) as a moral or conventional norm violation. In this process, we set up conventional game norms rather than prototypical conventional norms (i.e., not wearing pajamas to school or eating spaghetti with one’s hands), as the latter would have made it extremely challenging to control for visual differences. Further, we did not include a manipulation check of children’s understanding of the conditions. Thus, it might be argued that our participants did not understand the conventional scenario and only perceived the transgression in the Moral condition (but not the Conventional condition) as a norm violation. Although this might be a possibility, prior experimental work has used conventional game rules to create well-controlled comparisons between moral norms and conventional game norms (Schmidt et al., 2012; Hardecker et al., 2016). In addition, these studies show that 3-year-olds understand and respond differently to conventional game norms and moral norms (Schmidt et al., 2012; Hardecker et al., 2016). However, to further understand the moral/conventional distinction, future research should investigate physiological arousal differences across prototypical moral and conventional violations. Given substantial visual differences across such scenarios, researchers could utilize non-visual physiological tools (such as skin conductance) to explore the moral/conventional distinction and should additionally include manipulation checks to ensure that children have appropriately understood the various scenarios.

An important strength of the current study is that we can be confident that children’s internal arousal did not result from any emotional signals contained in the videos themselves. First, all of the actors in our videos talked in an entirely neutral tone, even during the transgression scenes. This was done to ensure that participants would not infer or “catch” any emotions from the actors, as even infants show increased arousal to others’ affect (Geangu et al., 2011). Second, rather than showing participants the whole transgression, we paused the transgression halfway in and ended the trial before the transgression was completed. Children thus did not see the victim’s or bystander’s reaction after the transgression. In these ways, we made sure that participants’ physiological arousal was due to their interpretation of the transgression as moral or conventional rather than any affective content in the videos.

Our phasic findings suggest that internal arousal accompanies the moral–conventional distinction from the earliest age at which the distinction is conceptually made (3 years of age). Although more research is needed to argue for a causal mechanism between affect and morality, the current study lays the groundwork for future research to explore how norms are moralized (or de-moralized). However, this is the first examination of physiological arousal following moral and conventional transgressions and, as such, must be interpreted with caution. One limitation to pupillometry, and indeed any physiological technique, is that it is not possible to disentangle which specific emotions caused the changes observed (Siegel et al., 2018). This points to another limitation to our interpretation of physiological arousal observed as a response to moral/conventional violations: young children and adults may show different moral emotions to transgressions. The research on moral emotions indicates that young children expect transgressors to feel happy after committing a transgression—also known as the happy-victimizer phenomenon (Krettenauer et al., 2008; Malti and Krettenauer, 2013). Extending these findings, it is possible that children in our study felt positively (rather than negatively) about the transgression/transgressor, whereas adults experienced more negative affect. However, due to the limitations inherent in interpreting pupil dilation, we cannot distinguish if children’s and adults’ physiological responses stem from the same or different affective valence. At the same time, there is, to our knowledge, no study relating changes in pupil dilation to positively valenced arousal. In contrast, to the degree that seeing others needing help is comparable to seeing others being harmed, young children’s pupil dilation increases in response to unfilled needs, which aligns with the interpretation that the changes in physiological arousal we found in the present study are more negative than positive in nature (Hepach et al., 2013).

It is also possible that factors other than affect may have influenced participants’ arousal. One important factor that causes changes in pupil size is luminance (Hepach and Westermann, 2016; Sirois and Brisson, 2014). However, we ensured that luminance was similar across participants by testing children and adults in the same room under the same lighting conditions and turning on the screen at least 20 min prior to participants’ arrival. Alternatively, increased cognitive load also increases pupil dilation (Ahern and Beatty, 1979; Naber and Nakayama, 2013; see Sirois and Brisson, 2014). It is possible that changes in pupil dilation observed in our study were due to information processing demands of moral and conventional norm violations and not due to affect. This possibility is partially ruled out by the fact that the final transgression scene was identical in both the moral and conventional violations. Nonetheless, future work should account for this explanation by using multiple physiological tools (e.g., skin conductance) to capture arousal independently from cognitive load.

Another factor that could cause changes in pupil size is increased attention to stimuli (Jackson and Sirois, 2009). For example, in violation of expectation paradigms, children show greater pupil dilation and also attend more to an improbable event compared to a probable event (Jackson and Sirois, 2009). It is possible that children and adults view moral transgressions as more improbable or irregular and may thus have attended to the moral violation more than the conventional violation. Note, however, that prior work with classroom observations indicates that moral and conventional violations are equally likely to occur (see Nucci and Turiel, 1978). Moreover, we accounted for this possibility in the present study by comparing the amount of time spent looking at the moral versus the conventional violation scene, and found that participants attended equally to both scenes. Because participants did not differ on the total duration of looking to the scenes but did differ on what they attended to in the scene (the victim in the Moral more than the bystander in the Conventional), our pupil dilation results are likely not due to differences in overall attention to the scenes but, rather, due to the differences in arousal elicited by the construal of those scenes as moral versus conventional transgressions.

In the present eye-tracking study, we found that moral rules arouse higher levels of affect than conventional rules not only among adults, as had previously been shown, but also among 3- and 4-year-old children. Both groups of children showed greater and immediate increase in internal arousal when an instrumental action caused harm compared to when the same action marked a conventional transgression. Participants of all ages also attended more to the victim of a moral transgression than the bystander in a conventional transgression. Thus, by 3 years of age, children not only distinguish moral from conventional transgressions based on the conceptual criteria that distinguish these domains but also are differently aroused by moral versus conventional transgressions. In sum, these results shed new light on the involvement of affect underlying the moral–conventional distinction in early childhood and open up new avenues for research on how these early affective and conceptual distinctions develop and influence each other.

## Data Availability Statement

All data and scripts for this study are available at: https://osf.io/pwknz.

## Ethics Statement

The studies involving human participants were reviewed and approved by the Institutional Review Board for Social and Behavioral Sciences at the University of Virginia. Written informed consent to participate in this study was provided by the participants, and their’ legal guardian/next of kin when appropriate.

## Author Contributions

MY, RH, and AV contributed to the conception and design of the study. MY and RH analyzed the data. MY wrote the manuscript with support from RH and AV. All authors discussed the results and contributed to the final manuscript.

## Conflict of Interest

The authors declare that the research was conducted in the absence of any commercial or financial relationships that could be construed as a potential conflict of interest.
