# Supplementary material for: Young Children and Adults Show Differential Arousal to Moral and Conventional Transgressions
Source: Front Psychol. 2020 Apr 17;11:548. doi: 10.3389/fpsyg.2020.00548 (PMC7212443; doi:10.3389/fpsyg.2020.00548)
Supplement: Supplementary file 1 [file Table_1.PDF]

Supplementary Material

1 Supplementary Figures and Tables

1.1 Supplementary Figure

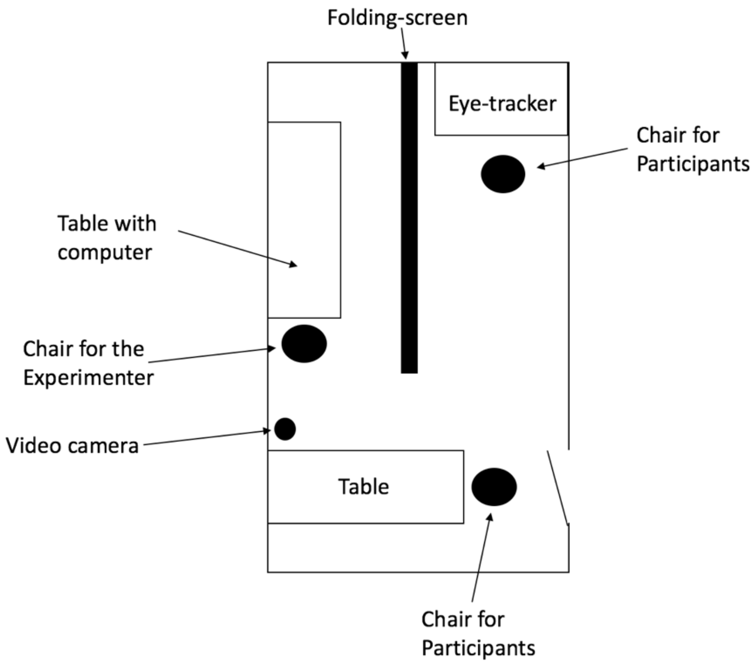

Figure S1. A bird's eye view of the experimental set-up.

1.2 Supplementary Table

Table S1. Number of participants per condition

	Moral		Conventional		
	Female	Male	Female	Male	Total
3-year-olds	8	7	7	10	32
4-year-olds	9	8	8	9	34
Adults	16	16	15	17	64

1.3 Supplementary Table

Table S2. Cell means for all analyses

Analysis	Moral			Conventional		
	3-year-olds	4-year-olds	Adults	3-year-olds	4-year-olds	Adults
Duration of looking (%)	20.80	19.51	14.32	15.38	13.83	14.67
	(6.38)	(8.71)	(7.50)	(5.74)	(9.53)	(8.17)
Pupil dilation: Phasic (Reaching only)	.07	.18	.07	.02	.06	.02
	(.12)	(.19)	(.14)	(.14)	(.15)	(.08)
Pupil dilation: Phasic (Transgression)	.07	.18	.07	.03	.03	-.02
	(.12)	(.19)	(.14)	(.13)	(.15)	(.13)
Pupil dilation: Tonic (Bubbles)	-.003	0.006	.02	.04	.04	- .003
	(.05)	(0.05)	(.07)	(.06)	(.04)	(.06)
Distribution task	1.33	1.31	1.00	1.53	1.47	1.10
(Flowers to transgressor)	(.82)	(.48)	(.52)	(.51)	(.64)	(.54)

2 Video Script

Participants in both conditions watched a 5-minute video consisting of two trials. One trial featured a drawing activity and the other trial featured a clay sculpting activity. The order of drawing and sculpting trials was counterbalanced across participants. Each video consisted of three action scenes: rule introduction, rule following, and rule transgression.

Moral: Paper

Scene 1 (Rule introduction; 0:00:01 - 0:00:56). The first scene in each trial started with three adults with neutral expressions seated next to each other at a table. The person in the middle was the moderator and the people on her left and right were the actors (Sally and Katie). The participant did not see or interact with any of these actors prior to or after the experiment. There was a drawing in front of each actor and Sally and Katie were working on their own drawings. The scene always began with the moderator introducing the two actors (Sally and Katie), by saying *"Look! Sally is drawing on her blue paper and Katie is drawing on her yellow paper."* The moderator waved at Sally (the transgressor) first, said, *"Hi Sally!"* then waved at Katie and said *"Hi Katie!"* Both actors smiled, said "Hi," and waved when their names were mentioned. The moderator then said, "*Have you been working on your drawings?"* and both actors nodded. *"Great. You can continue working on your drawings* [points to the two drawings on the table]. *But you should only work on your own drawings. You can of course tear up your own drawing if you like, but you should be careful not to tear the other person's drawing."*

Attention grabbing transition scene (0:00:56 - 0:01:06). Purple bubbles appeared on the screen.

Conventional: Paper

Scene 1 (Rule introduction; 0:00:01 - 0:00:56). The first scene in each trial started with three adults with neutral expressions seated next to each other at a table. The person in the middle was the moderator and the people on her left and right were the actors (Sally and Katie). The participant did not see or interact with any of these actors prior to or after the experiment. There were two drawings in the center of the table, and the two actors were looking straight ahead at the camera. The scene always began with the moderator introducing the two actors (Sally and Katie), by saying *"Look! It's Sally and Katie."* The moderator waved at Sally (the transgressor) first, said, *"Hi Sally!"* then waved at Katie and said *"Hi Katie!"* Both actors smiled, said "Hi," and waved when their names were mentioned. The moderator then said, *"You're both going to play a game. Are you ready?"* and both actors nodded. She then said, *"Look there are some drawings. The game is to tear all the drawings on the table. The way to tear the drawings is to take a ruler* [points to the two rulers on the table]*, put it on top of the drawings and then tear along the ruler. You are not allowed to tear the drawings just with your hands, you can only tear them using the ruler."*

Attention grabbing transition scene (0:00:56 - 0:01:06). Purple bubbles appeared on the screen.

Moral continued

Scene 2 (Rule following; 0:01:06 - 0:01:51). Sally, Katie, and the moderator appeared on the screen, sitting at their previous location. Sally (with a neutral facial expression) picked up her drawing and tore it up with her hands. Katie and the moderator both watched her tear it with a neutral expression. Then, Katie (with a neutral facial expression) picked up her drawing and tore it up with her hands. Sally and the moderator both watched her tear it with a neutral expression. The moderator then said in a neutral tone, *"Great! You both worked with your own drawing! Remember, if you want to tear your own drawing, that's fine. But you may not tear the other person's drawing."*

Attention grabbing transition scene (0:01:51 - 0:02:01). Purple bubbles appeared on the screen.

Scene 3 (Rule transgression; 0:02:01 - 0:02:25). This scene was identical to the corresponding scene in the Conventional condition. The moderator was now absent from the scene. Sally reached over to the drawing that was closer to Katie, and the scene froze for 5s. She then placed it in front of her, and said, *"I'm going to tear this now"* in a neutral tone. Sally then started to tear the drawing using her hands only. The scene froze halfway through Sally tearing and Katie watching it.

Attention grabbing transition scene (0:02:25 - 0:02:35). Purple bubbles appeared on the screen.

Conventional continued

Scene 2 (Rule following; 0:01:06 - 0:01:51). Sally, Katie, and the moderator appeared on the screen, sitting at their previous location. Sally (with a neutral facial expression) picked up the drawing across from her, and tore it up using her ruler. Katie and the moderator both watched her tear it with a neutral expression. Then, Katie (with a neutral facial expression) picked up the drawings across from her, and tore it up using her ruler. Sally and the moderator both watched her tear it with a neutral expression. The moderator then said in a neutral tone, *"Great! You both played the game right! Remember, you are not allowed to tear the drawing just with your hands. You can only tear them using the ruler."*

Attention grabbing transition scene (0:01:51 - 0:02:01). Purple bubbles appeared on the screen.

Scene 3 (Rule transgression; 0:02:01 - 0:02:25). This scene was identical to the corresponding scene in the Moral condition. The moderator was now absent from the scene. Sally reached over to the drawing closer to Katie, and the scene froze for 5s. She then placed it in front of her, and said, *"I'm going to tear this now"* in a neutral tone. Sally then started to tear the drawing using her hands only. The scene froze halfway through Sally tearing and Katie watching it.

Attention grabbing transition scene (0:02:25 - 0:02:35). Purple bubbles appeared on the screen.

Moral: Sculpture

Scene 1 (Rule introduction; 0:00:01 - 0:00:56). The first scene in each trial started with three adults with neutral expressions seated next to each other at a table. The person in the middle was the moderator and the people on her left and right were the actors (Sally and Katie). The participant did not see or interact with any of these actors prior to or after the experiment. There was a sculpture in front of each actor and Sally and Katie were working on their own sculptures. The scene always began with the moderator introducing the two actors (Sally and Katie), by saying *"Look! Sally is working with her blue playdough and Katie is working with her yellow playdough."* The moderator waved at Sally (the transgressor) first, said, *"Hi Sally!"* then waved at Katie and said *"Hi Katie!"* Both actors smiled, said "Hi," and waved when their names were mentioned. The moderator then said, "*Have you been working on your sculptures?"* and both actors nodded. *"Great. You can continue working on your sculptures* [points to the two sculptures on the table]. *But you should only work on your own sculpture. You can of course squish up your own sculpture if you like, but you should be careful not to squish the other person's sculpture."*

Attention grabbing transition scene (0:00:56 - 0:01:06). Purple bubbles appeared on the screen.

Conventional: Sculpture

Scene 1 (Rule introduction; 0:00:01 - 0:00:56). The first scene in each trial started with three adults with neutral expressions seated next to each other at a table. The person in the middle was the moderator and the people on her left and right were the actors (Sally and Katie). The participant did not see or interact with any of these actors prior to or after the experiment. There were two sculptures in the center of the table, and the two actors were looking straight ahead at the camera. The scene always began with the moderator introducing the two actors (Sally and Katie), by saying *"Look! It's Sally and Katie."* The moderator waved at Sally (the transgressor) first, said, *"Hi Sally!"* then waved at Katie and said *"Hi Katie!"* Both actors smiled, said "Hi," and waved when their names were mentioned. The moderator then said, *"You're both going to play a game. Are you ready?"* and both actors nodded. *"Look there are some sculptures. The game is to squish all the sculptures and flatten them out. The way to squish the sculptures is to take a block* [points to the two blocks on the table], *put it on top of the sculptures and press down. You are not allowed to squish the sculptures just with your hands, you can only squish them using the block."*

Attention grabbing transition scene (0:00:56 - 0:01:06). Purple bubbles appeared on the screen.

Moral continued

Scene 2 (Rule following; 0:01:06 - 0:01:51). Sally, Katie, and the moderator appeared on the screen, sitting at their previous location. Sally (with a neutral facial expression) picked up her sculpture and squished it up with her hands. Katie and the moderator both watched her squish it with a neutral expression. Then, Katie (with a neutral facial expression) picked up her sculpture and squished it up with her hands. Sally and the moderator both watched her squish it with a neutral expression. The moderator then said in a neutral tone *"Great! You both worked with your own sculptures! Remember, if you want to squish your own sculpture, that's fine. But you may not squish the other person's sculpture."*

Attention grabbing transition scene (0:01:51 - 0:02:01). Purple bubbles appeared on the screen.

Scene 3 (Rule transgression; 0:02:01 - 0:02:25). This scene was identical to the corresponding scene in the Conventional condition. The moderator was now absent from the scene. Sally reached over to the sculpture that was closer to Katie, and the scene froze for 5s. She then placed it in front of her, and said, *"I'm going to squish this now"* in a neutral tone. Sally then started to squish the sculpture using her hands only. The scene froze halfway through Sally squishing and Katie watching it.

Attention grabbing transition scene (0:02:25 - 0:02:35). Purple bubbles appeared on the screen.

Conventional continued

Scene 2 (Rule following; 0:01:06 - 0:01:51). Sally, Katie, and the moderator appeared on the screen, sitting at their previous location. Sally (with a neutral facial expression) picked up the sculpture across from her, and squished it using her block. Katie and the moderator both watched her squish it with a neutral expression. Then, Katie (with a neutral facial expression) picked up the sculpture across from her, and squished it using her block. Sally and the moderator both watched her squish it with a neutral expression. The moderator then said in a neutral tone, *"Great! You both played the game right! Remember, you are not allowed to squish the sculptures just with your hands. You can only squish them using the block."*

Attention grabbing transition scene (0:01:51 - 0:02:01). Purple bubbles appeared on the screen.

Scene 3 (Rule transgression; 0:02:01 - 0:02:25). This scene was identical to the corresponding scene in the Moral condition. The moderator was now absent from the scene. Sally reached over to the sculpture that was closer to Katie, and the scene froze for 5s. She then placed it in front of her, and said, *"I'm going to squish this now"* in a neutral tone. Sally then started to squish the sculpture using her hands only. The scene froze halfway through Sally squishing and Katie watching it.

Attention grabbing transition scene (0:02:25 - 0:02:35). Purple bubbles appeared on the screen.
